# Cognitive performance and functional outcomes of carriers of pathogenic copy number variants: analysis of the UK Biobank

**DOI:** 10.1192/bjp.2018.301

**Published:** 2019-05

**Authors:** Kimberley M. Kendall, Matthew Bracher-Smith, Harry Fitzpatrick, Amy Lynham, Elliott Rees, Valentina Escott-Price, Michael J. Owen, Michael C. O'Donovan, James T.R. Walters, George Kirov

**Affiliations:** 1 Wellcome Trust Clinical Research Fellow, MRC Centre for Neuropsychiatric Genetics and Genomics, Cardiff University, UK; 2 PhD Student, MRC Centre for Neuropsychiatric Genetics and Genomics, Cardiff University, UK; 3 Medical Student, School of Medicine, Cardiff University, UK; 4 Research Associate, MRC Centre for Neuropsychiatric Genetics and Genomics, Cardiff University, UK; 5 Professor, Dementia Research Institute, Cardiff University, UK; 6 Director, MRC Centre for Neuropsychiatric Genetics and Genomics, Cardiff University; Director/Clinical Professor, Division of Psychological Medicine and Clinical Neuroscience, Cardiff University; and Emeritus Director, Neuroscience and Mental Health Research Institute, Cardiff University, UK; 7 Deputy Director, Division of Psychological Medicine and Clinical Neurosciences, MRC Centre for Neuropsychiatric Genetics and Genomics, Cardiff University, UK; 8 Professor, Division of Psychological Medicine and Clinical Neurosciences, MRC Centre for Neuropsychiatric Genetics and Genomics, Cardiff University, UK; 9 Professor, Division of Psychological Medicine and Clinical Neurosciences, MRC Centre for Neuropsychiatric Genetics and Genomics, Cardiff University, UK

**Keywords:** CNV, cognitive, UK Biobank, Townsend Deprivation Index, schizophrenia

## Abstract

**Background:**

Rare copy number variants (CNVs) are associated with risk of neurodevelopmental disorders characterised by varying degrees of cognitive impairment, including schizophrenia, autism spectrum disorder and intellectual disability. However, the effects of many individual CNVs in carriers without neurodevelopmental disorders are not yet fully understood, and little is known about the effects of reciprocal copy number changes of known pathogenic loci.

**Aims:**

We aimed to analyse the effect of CNV carrier status on cognitive performance and measures of occupational and social outcomes in unaffected individuals from the UK Biobank.

**Method:**

We called CNVs in the full UK Biobank sample and analysed data from 420 247 individuals who passed CNV quality control, reported White British or Irish ancestry and were not diagnosed with neurodevelopmental disorders. We analysed 33 pathogenic CNVs, including their reciprocal deletions/duplications, for association with seven cognitive tests and four general measures of functioning: academic qualifications, occupation, household income and Townsend Deprivation Index.

**Results:**

Most CNVs (24 out of 33) were associated with reduced performance on at least one cognitive test or measure of functioning. The changes on the cognitive tests were modest (average reduction of 0.13 s.d.) but varied markedly between CNVs. All 12 schizophrenia-associated CNVs were associated with significant impairments on measures of functioning.

**Conclusions:**

CNVs implicated in neurodevelopmental disorders, including schizophrenia, are associated with cognitive deficits, even among unaffected individuals. These deficits may be subtle but CNV carriers have significant disadvantages in educational attainment and ability to earn income in adult life.

**Declaration of interest:**

None.

Copy number variants (CNVs) are deletions or duplications of >1000 DNA base pairs, resulting in altered dosage of the affected sequence.^1^^,^^2^ Rare (frequency <1%) CNVs are associated with risk of neurodevelopmental disorders, including intellectual disability, autism spectrum disorder, attention-deficit hyperactivity disorder and schizophrenia, disorders characterised by varying degrees of cognitive impairment.^3^^–^^8^ Carriers of such CNVs can have cognitive impairment even if they do not have such diagnoses. Previous work, including our study of the first ~150 000 individuals genotyped in UK Biobank, has shown that unaffected individuals carrying neurodevelopmental CNVs, as a group, perform on cognitive tests intermediately between CNV non-carriers and individuals with schizophrenia, and have lower educational attainment and occupations requiring less training, compared with non-carriers.^9^^–^^11^ As a group, rare deletion CNVs have also been associated with a decrease in performance IQ in general population cohorts.^12^ Our previous work lacked power to examine the effect of individual CNVs. Since then, genotype data on a further ~350 000 individuals have been released. The aim of the present study was to examine the effects of individual CNVs on cognitive function and on more general measures of functioning, including educational performance and ability to earn income. Given pathogenic CNVs are thought to exert their phenotypic effects via changes in gene dosage, we also included in the analysis the reciprocal deletions/duplications of known pathogenic CNVs, even if their role on cognition has not been identified before.

## Method

### Participants

The UK Biobank recruited ~500 000 individuals aged 40–69 years (54% female) between 2006 and 2010. Participants underwent phenotypic and cognitive testing at UK Biobank assessment centres, and provided demographic, socioeconomic and health data. Subgroups also completed follow-up testing in person and online. Written informed consent was obtained from all participants by the UK Biobank. All procedures involving human participants were approved by the North West Multi-Centre Ethics Committee (approval number 11/NW/0382). Data were released to Cardiff University under application number 14421 ‘*Identifying the spectrum of biomedical traits in adults with pathogenic copy number variants (CNVs)*’. The authors assert that all procedures contributing to this work comply with the ethical standards of the relevant national and institutional committees on human experimentation and with the Helsinki Declaration of 1975, as revised in 2008.

To limit the analysis to unaffected individuals, we excluded 1021 individuals diagnosed with intellectual disability, autism spectrum disorder or schizophrenia, (self-reported or diagnosed during hospital admissions).

### CNV calling and annotation

Samples were genotyped at Affymetrix Research Services Laboratory (Santa Clara, California, USA) on two Affymetrix arrays: ~50 000 on the UK BiLEVE Array and ~450 000 on the UK Biobank Axiom Array. Quality control filters included genotypic call rate <0.96, waviness factor of <−0.03 and >0.03, >30 CNVs per person and log R ratio s.d. of >0.35. From 488 415 people with genotypic data, 25 061 were excluded for failing these quality control filters. We further restricted analyses to individuals of self-reported White British or Irish ancestry, for a total of 420 247 individuals included in the analysis. Participants passing quality control had an average of 6.57 CNVs called with ≥10 SNP probes. Our CNV calling pipeline is described in detail elsewhere.^10^ Briefly, we downloaded raw intensity CEL files and processed them with Affymetrix Power Tools (version 1.18.0 for Linux/OS X, Affymetrix, US www.affymetrix.com/estore/partners_programs/programs/developer/tools/powertools.affx) and PennCNV.^13^ We compiled an initial list of 92 CNV regions that lead to genomic disorders, or are implicated in intellectual disability, autism spectrum disorder and congenital malformations, and their reciprocal deletions or duplications, from two widely accepted sources.^5^^,^^14^ Most of these CNVs have been shown to statistically increase risk for developmental delay;^5^ however, the list includes the reciprocal deletions/duplications of confirmed pathogenic CNVs, even if the evidence for their pathogenicity in unclear. Details on the 92 CNV loci are available in our previous publications on the UK Biobank.^10^^,^^15^ CNVs with fewer than five observations for any of the seven cognitive tests, as well as three CNV loci that produced frequent calls on arrays that failed quality control (false positives), were excluded from analyses, resulting in 33 CNVs taken forward for association analyses (Table 1). Five of the 12 known schizophrenia-associated CNVs^7^ had to be excluded from analyses, as fewer than five carriers had completed one or more of the tests: deletions at 15q13.3 (total *n* = 41), 22q11.2 (*n* = 8) and 3q29 (*n* = 7), and duplications at the Williams–Beuren (*n* = 13) and the Prader–Willi syndrome regions (*n* = 17). We only report their associations with the general measures of functioning, where data is available on most participants (*Discussion*).

**Table 1:** CNVs examined in analyses

CNV locus	Genomic location (Mb)	Number of genes in CNV	Number of CNV carriers (%)	Number of significant results	Average effect size	Penetrance (%)
^a^TAR del	chr1:145,39–145,81	17	75 (0.018)	0	−0.11	17
^a^TAR dup	chr1:145,39–145,81	17	436 (0.1)	5	−0.12	7
^b^1q21.1 del	chr1:146,53–147,39	9	112 (0.027)	7	−0.27	28
^b^1q21.1 dup	chr1:146,53–147,39	9	176 (0.042)	5	−0.08	14
^b^*NRXN1* del	chr2:50,14–51,26	1	162 (0.039)	4	−0.19	13
^a^2q11.2 del	chr2:96,74–97,68	22	31 (0.007)	1	−0.13	13
2q13 del (*NPHP1*)	chr2:110,86–110,98	3	2446 (0.58)	0	−0.01	4
2q13 dup (*NPHP1*)	chr2:110,86–110,98	3	1972 (0.47)	0	0.01	9
^a^2q13 del	chr2:111,39–112,01	3	53 (0.013)	3	−0.16	17
^a^2q13 dup	chr2:111,39–112,01	3	71 (0.017)	0	−0.02	6
2q21.1 dup	chr2:131,48–131,93	5	59 (0.014)	0	0.07	9
^a^10q11.21q11.23 del	chr10:49,39–51,06	19	57 (0.014)	4	−0.18	7
10q11.21q11.23 dup	chr10:49,39–51,06	19	41 (0.01)	0	0.03	11
13q12 del (*CRYL1*)	chr13:20,98–21,10	2	379 (0.09)	1	0.01	6
13q12.12 del	chr13:23,56–24,88	10	84 (0.02)	2	−0.16	4
13q12.12 dup	chr13:23,56–24,88	10	233 (0.055)	0	−0.01	3
^b^15q11.2 del	chr15:22,81–23,09	5	1661 (0.39)	9	−0.15	8
^a^15q11.2 dup	chr15:22,81–23,09	5	2039 (0.48)	5	−0.03	5
15q11q13 dup breakpoints 3 and 4	chr15:29,16–30,38	4	53 (0.013)	2	−0.18	4
15q13.3 dup	chr15:31,08–32,46	8	240 (0.057)	6	−0.09	8
15q13.3 dup (*CHRNA7*)	chr15:32,02–32,46	1	3023 (0.72)	1	0.01	5
^a^16p13.11 del	chr16:15,51–16,29	7	131 (0.031)	6	−0.26	17
^b^16p13.11 dup	chr16:15,51–16,29	7	825 (0.2)	7	−0.06	7
^b^16p12.1 del	chr16:21,95–22,43	8	245 (0.058)	7	−0.10	11
16p12.1 dup	chr16:21,95–22,43	8	198 (0.047)	0	−0.09	3
^a^16p11.2 distal del	chr16:28,82–29,05	11	58 (0.014)	7	−0.41	23
^a^16p11.2 distal dup	chr16:28,82–29,05	11	136 (0.032)	4	−0.20	11
^a^16p11.2 del	chr16:29,65–30,20	30	104 (0.025)	7	−0.34	38
^b^16p11.2 dup	chr16:29,65–30,20	30	134 (0.032)	8	−0.41	29
17p12 del (HNPP)	chr17:14,14–15,43	8	237 (0.056)	0	0.01	5
17p12 dup (CMT1A)	chr17:14,14–15,43	8	124 (0.030)	2	−0.04	9
^a^17q12 dup	chr17:34,81–36,22	17	100 (0.024)	4	−0.18	14
^a^22q11.2 dup	chr22:19,04–21,47	61	279 (0.066)	11	−0.32	14

The number of significant results is shown, using a threshold of false discovery rate of 0.05. The average effect size is the mean of the seven cognitive tests coefficients for that CNV from regression analyses (a lower number indicates worse impairment). Penetrance data are based on Kirov *et al*,^16^ with control frequencies updated by UK Biobank data and indicate the cumulative risk of CNV carriers to develop schizophrenia, autism spectrum disorder or intellectual disability. CNV, copy number variant; del, deletion; chr, chromosome; dup, duplication; HNPP, hereditary neuropathy with liability to pressure palsies.

a. Neurodevelopmental CNV.

b. Schizophrenia-associated CNV.

### Cognitive tests

Participants completed cognitive tests at UK Biobank recruitment centres (http://biobank.ctsu.ox.ac.uk/crystal/label.cgi?id=100026) and a subgroup completed follow-up testing online (http://biobank.ctsu.ox.ac.uk/crystal/label.cgi?id=116). We analysed tests performed by at least ~20% of participants. Where the same test was performed at baseline and follow-up, we chose the occasion when the test was completed by a higher number of people. In total, seven cognitive tests were examined for association with individual CNVs. Before association analyses, data were normalised, where required, and converted to *Z*-scores as described previously.^10^

#### Pairs Matching Test

The Pairs Matching Test is a test of episodic memory and was completed by 419 861 individuals included in analyses. Participants were shown 6 pairs of cards for 3 s and then asked to identify matching pairs. We used the total number of errors for participants who completed the test as our outcome measure and applied a log + 1 transformation to these data.

#### Reaction Time Test

The Reaction Time Test is a test of simple processing speed and was completed by 417 461 individuals included in analyses. Participants played 12 rounds of the game ‘Snap’, where they had to click a button as quickly as possible when shown matching cards. We used the mean reaction time to correct responses as our outcome measure. A log transformation was applied to these data.

#### Fluid Intelligence Test

The Fluid Intelligence Test is a test of reasoning and problem solving and was completed at baseline by 134 309 individuals included in analyses. Participants completed as many verbal and numerical reasoning questions as they could within 2 min. We used the total number of correct answers as the outcome measure.

#### Digit Span Test

The Digit Span Test is a test of numeric working memory and was completed at an online follow-up by 92 379 individuals included in analyses. Participants were required to try to remember progressively longer numbers and enter them once the number had disappeared from the screen. We used the maximum number of digits remembered as our outcome measure.

#### Symbol Digit Substitution Test

The Symbol Digit Substitution Test is a test of complex processing speed and was completed at the online follow-up by 102 042 individuals included in analyses. Participants were required to match as many numbers to symbols as they could in a given time. We used the number of correct substitutions as our outcome measure, excluding outliers (<3 and >36 substitutions). The remaining scores were normally distributed and did not require transformation.

#### Trail Making Tests A and B

The Trail Making Tests A and B are tests of visual search speed, scanning, speed of processing, mental flexibility and executive functioning. They were completed by 90 106 individuals at online follow-up. Participants were required to connect circles according to numbers (test A) and alternating letters and numbers (test B). We used the time taken to complete these tests as our outcome measure. A log transformation was applied to these data.

### General measures of functioning

Cognitive performance affects the educational attainment, functioning and earning potential of people throughout their lives.^17^^–^^19^ We chose to analyse four measures of functioning, with data available on most participants: educational qualifications, occupation, household income and Townsend Deprivation Index.^20^

#### Qualifications

We used the highest qualification an individual had achieved (e.g. university/college degree, A-levels). After excluding participants with only ‘other professional qualifications’ and those who did not answer the question, we retained data for 394 575 participants, as described previously.^10^

#### Occupation

We used the nine bands coding for occupations, based on the required training or academic skills, as defined by the UK Office of National Statistics.^21^ After excluding those who declined to answer, or whose occupations could not be fitted into the nine categories, we retained data for 257 788 participants.

#### Household income

Annual income was categorised by the UK Biobank in five bands, as follows: <£18,000, £18,000–30,999, £31,000–51,999, £52,000–100,000 and >£100,000. Information was available on 361 812 included participants.

#### Townsend Deprivation Index

This is an index of social deprivation. Participants were assigned deprivation scores based on their postal code at the time of recruitment. The score is based on four variables: households without a car, overcrowded households, households not owner occupied and persons unemployed. Information was available on 419 756 included participants.

### Statistical analyses

Analyses were performed in R (v3.3.2). For all cognitive tests and the Townsend Deprivation Index we used linear regression analyses (glm) with cognitive score as the dependent variable. We used CNV carrier status as the independent variable and included as covariates age at the time of assessment, gender and array type (BiLEVE or Axiom), and for the cognitive tests, measures of medical comorbidity, psychotropic medication and alcohol intake, which all negatively affected the cognitive tests. As a proxy for medical comorbidity, we used the number of hospital admissions (log + 1 transformed); for psychotropic medication intake, we created four separate variables for antipsychotics, antidepressants, benzodiazepines and anti-epileptics, and for alcohol intake we used the self-reported alcohol consumption frequency. Results are presented as unstandardised regression coefficients (that equate to *Z*-score differences between CNV carriers and non-carriers). Results for educational qualifications, occupation and household income, which were analysed in ordinal regression analyses, are expressed as the exponential of the odds of carriers being in a different band (e.g. in a lower income bracket). For ease of interpretation, the directions of all effects were adjusted in all tables and figures, so that a negative sign always implies worse performance or outcome, e.g. longer time to complete a test, fewer digits remembered, lower income and higher Townsend Deprivation Index. To evaluate the significance of results, we used the Benjamini–Hochberg false discovery rate (FDR) method for *P*-value correction, as multiple true positive results were expected. We accepted an FDR of 0.05 as our significance threshold (i.e. we expect 5% of the results below that threshold to be false positives).^22^

## Results

**Fig. 1:**
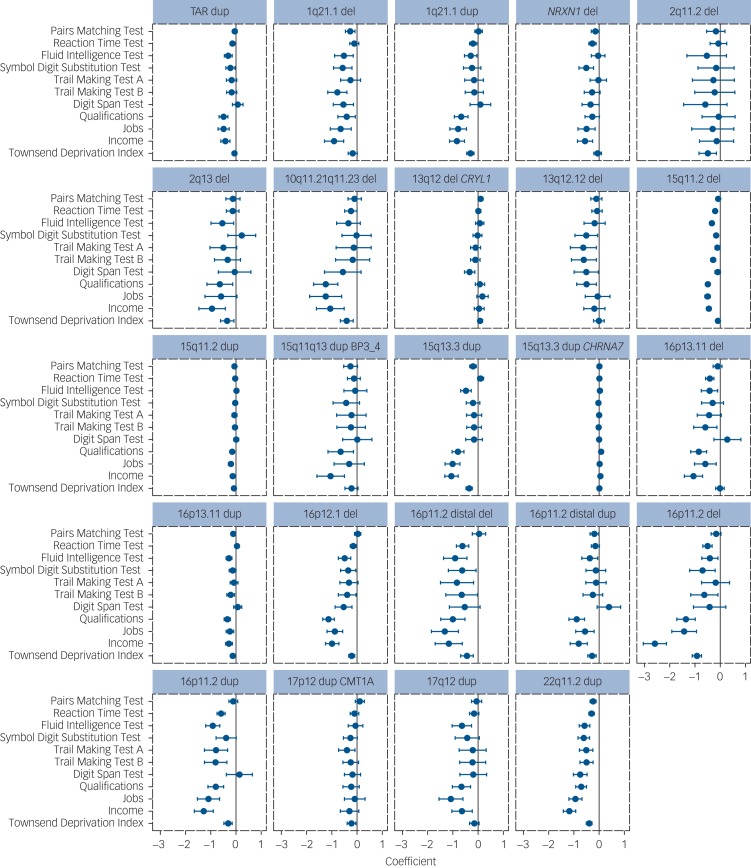
Results of association analyses of pathogenic loci with seven cognitive tests and four measures of functioning for the 24 CNVs with at least one significant result. The direction of effect is adjusted so that poorer performance/functioning is always indicated with a negative sign (to the left of the zero-point vertical lines). The coefficients and 95% CIs are derived from linear regression analyses, except for income, occupations and educational qualifications, which were analysed with ordinal regression analysis. Higher definition images for the effect sizes of all 33 CNVs are presented in Supplementary Figure 1. CNV, copy number variant; del, deletion; dup, duplication.

The majority of tested CNVs (24 out of 33) were significantly associated with at least one cognitive performance test or measure of functioning (Table 1, Fig. 1). A total of 118 of the 363 individual associations (32.5%) were significant at FDR <0.05. Full details are given in Supplementary Table 1 and Figure 1 available at https://doi.org/10.1192/bjp.2018.301.

Of the 118 significant results, 117 were in the direction of reduced performance or function. The average effect size for the seven cognitive tests was only −0.13 (range, −0.41 to 0.07), with the worst performance among carriers of 16p11.2 deletions and duplications, 16p11.2 distal deletions and 22q11.2 duplications (range, −0.34 to −0.41). The single trend toward improved performance (15q13.3 *CHRNA7* duplication with qualifications) was our least significant result, compatible with it being one of the expected 5% false positives. This suggests that no CNV from this list enhances cognitive function and there are no mirror phenotypes caused by deletions and duplications at the same locus (i.e. deletion impairing cognitive function and duplication enhancing it), something shown for some physical traits such as height and weight among carriers of CNVs at the 16p11.2 locus.^23^^,^^24^ The 15q13.3 duplication had six significant associations. The reciprocal deletion at this locus is a confirmed schizophrenia/intellectual disability/autism spectrum disorder risk locus, but the duplication has not yet been statistically confirmed as a neurodevelopmental CNV.^5^ Our results suggest that it is probably also a true intellectual disability/autism spectrum disorder locus despite failing to reach statistical support in the previous analysis.^5^ To test the robustness of our findings, we performed permutation analysis of the six significant results by randomly shuffling the carrier status of individuals 10 000 times and repeating the regression analysis. All six results were robust to permutation testing (Supplementary Table 1).

## Discussion

Associations between neurodevelopmental CNVs, as a group, and impaired cognitive performance among individuals without neurodevelopmental disorders were expected given previous work by us and others,^9^^–^^11^ and their associations with disorders characterised by varying degrees of cognitive impairment.^3^^,^^5^^–^^8^ We have now extended those findings to investigate the effects at the level of individual CNVs among unaffected adults from the general population. The effects tended to be modest, but the large sample size provided sufficient statistical power to detect existing differences. The range of impairments in carriers of deletions at *NRXN1* and 15q11.2, and duplications at 22q11.2 was larger than anticipated. In contrast, no significant cognitive or functional impairment was observed in carriers of nine CNVs: deletions/duplications at 2q13 (*NPHP1*), TAR deletions, duplications at 2q13, 2q21.1, 10q11.21q11.23, 13q12.12 and 16p12.1, and deletions at 17p12 (a CNV that causes the peripheral neurological disorder hereditary neuropathy with liability to pressure palsies).

### Cognitive impairment, penetrance of CNVs and selection

**Fig. 2:**
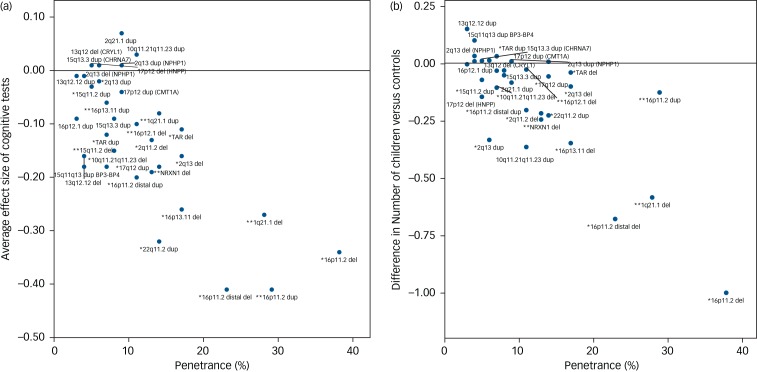
Correlation between the penetrance of CNVs for neurodevelopmental disorders (including intellectual disability, autism spectrum disorder and schizophrenia) and (a) the average effect sizes on seven cognitive tests (Pearson correlation, 0.74) and (b) the average number of children of CNV carriers (Pearson correlation, 0.78). * indicates neurodevelopmental CNV and ** indicates schizophrenia CNV. CNV, copy number variant.

The degree of cognitive impairment caused by each CNV can be approximated by their mean effect sizes from all seven tests. We hypothesised that there should be a positive correlation between the degree of cognitive impairment and the penetrance of these CNVs for the development of schizophrenia and other neurodevelopmental disorders. We used penetrance estimates from previous work,^16^ updated to include controls from the current UK Biobank cohort (Table 1). There was a strong correlation between the penetrance of CNVs for neurodevelopmental disorders and the average effect size of the seven cognitive tests among unaffected adult carriers (Pearson's correlation, 0.74; *P* = 10^−6^; Fig. 2a). Previous work reported a strong association between IQ and the probability at which CNV deletions occur *de novo*.^23^ This *de novo* probability reflects the strength of natural selection pressure on CNV carriers (*de novo* CNVs are filtered out by natural selection). There is a high correlation between penetrance for neurodevelopmental disorders and selection pressure, as we showed previously.^16^ Thus both papers suggest that cognitive function is a leading factor influencing the strength of selection pressure on CNV carriers. The selection pressure can be measured by comparing the number of surviving offspring from individuals with and without CNVs. This is not possible with the current data, as negative selection operates most strongly on persons affected with intellectual disability, autism spectrum disorder and schizophrenia, who are excluded from this analysis. For completeness, we provide data on the average number of offspring in unaffected CNV carriers, compared with the controls’ average of 1.8, after controlling for gender and age (Supplementary Table 2). Ten CNVs are associated with statistically significant reductions in the number of offspring, with the largest difference found for carriers of 16p11.2 deletions, who have on average one child fewer compared with controls. The reduction in the number of offspring also correlates highly with the penetrance of CNVs for neurodevelopmental disorders (Pearson's correlation, 0.78; *P* = 10^−7^; Fig. 2b). These results indicate that unaffected carriers of certain CNVs might also have deficits in socialising and forming families, likely due to a combination of cognitive, medical and behavioural problems.

### CNVs and functioning

The available cognitive tests provide a cross-sectional measure of a limited set of skills, and performance can be affected by random factors, such as distraction during tests performed on home computers. In contrast, overall school results, occupational attainment, the ability to earn an income and the degree of social deprivation in middle age represent overall real-world functional outcomes averaged across life. These measures of overall functioning showed an even higher rate of significant deficits: 70 out of 132 comparisons (53%) were significant at FDR = 0.05 (Fig. 1, Supplementary Table 1).

The cognitive effects of neurodevelopmental CNVs are an obvious potential mediator for the relatively poor overall functioning of carriers. However, they might not be the only factor. To explore this, we used the Fluid Intelligence Test score (the test with the strongest correlation with measures of functioning) as a covariate in regression analyses of measures of functioning. We then compared results with and without Fluid Intelligence Test score as a covariate (Supplementary Tables 3 and 4). The effect size for associations of neurodevelopmental CNVs with household income were modestly reduced, and they were almost unchanged for the Townsend Deprivation Index. This finding is consistent with the relatively low correlations between some of the cognitive test results and measures of functioning (Supplementary Table 5). This indicates that cognitive performance cannot account for the entire effect of neurodevelopmental CNVs on functioning. Of course, we cannot exclude the possibility that adjustment for better measures of cognition, including social cognition, might account for a greater proportion of the variance in these outcome measures, but it is intuitively likely that non-cognitive factors are also important. For example, associated medical problems can also reduce the ability to earn an income. One factor that is unlikely to play a role is the direct stigma of having a genetic disorder, as these CNVs would not have been screened for during the early life of the UK Biobank participants, when genetic testing is normally performed. It is, however, possible that some carriers display subtle physical features that might have resulted in discrimination; for example, mild dysmorphic features. Regardless of the cause, CNV carrier status should not be viewed as a deterministic factor in functional outcomes, as poor physical health and social disadvantage can be addressed and treated.

### Schizophrenia-associated CNVs

**Fig. 3:**
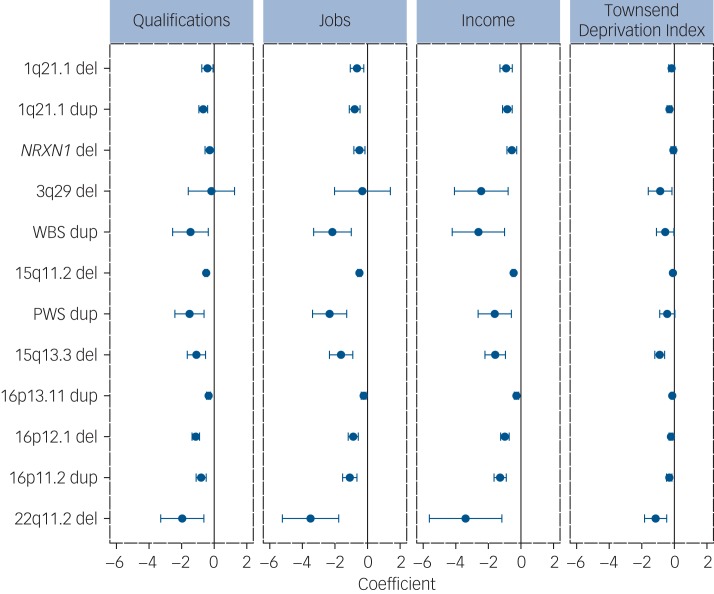
Analysis of associations between 12 schizophrenia-associated CNVs and measures of functioning. The ‘coefficients’ indicate the effect sizes and 95% CIs of the effect sizes from the regression analysis (*Methods*). All effects are in the direction of worse functioning (to the left of the zero-point vertical lines). CNV, copy number variant; del, deletion; dup, duplication.

CNVs robustly associated with risk for schizophrenia were consistently associated with impaired cognitive performance and measures of functioning: the seven CNVs analysed (out of 12 confirmed loci)^7^^,^^8^ were significantly associated with between four and nine tests/measures (Table 1). The remaining five CNVs (deletions at 15q13.3, 22q11.2 and 3q29, and duplications at the Williams-Beuren and Prader-Willi syndrome regions) are among the most highly penetrant for neurodevelopmental disorders,^16^ but were too rare to analyse. For completeness, we present the data for all 12 schizophrenia-associated CNVs for association with measures of functioning, which were available for most individuals. All 12 CNVs were associated with reduced functioning, with 45 out of the 48 comparisons reaching nominal levels of statistical significance (Fig. 3, Supplementary Table 6).

The UK Biobank consists of individuals who are healthier and have higher levels of education than the general population because of self-selection bias.^25^ It is also relatively depleted of individuals with neurodevelopmental disorders: e.g. only 52 individuals passing quality control had a known diagnosis of autism spectrum disorder and 802 had a diagnosis of schizophrenia, instead of the expected ~1% each (~4000 persons) under no selection bias. We excluded such individuals from the analyses. Consequently, our analyses underestimate the effect sizes of the more pathogenic CNVs which are rare in this population, as the most affected carriers are not ascertained. We show that in this population, certain neurodevelopmental CNVs are also associated with significant impairments in cognition. The effects on the level of household income among carriers of schizophrenia-associated CNVs are particularly striking, given that these carriers do not have such diagnoses (Fig. 3). These effects are not fully mediated by the measures of cognitive function available in the UK Biobank and cannot be explained by any stigma associated with a genetic disorder.

### Limitations

One limitation in the data is in the type of cognitive tests used: to maximise adherence, the chosen cognitive tests had to be administered quickly. They cannot test all types of cognitive abilities in great detail. In addition, very few individuals had completed all seven cognitive tests, making it impractical to calculate a general intelligence factor (*g*), as power would be severely affected. Instead, we decided to report all seven tests separately, thus increasing multiple testing. The ‘average effect size’ that we use (Table 1, Fig. 2a) is an imperfect measure because it assumes equal weight for each test, and because carriers of different CNVs did not complete identical sets of tests. These problems also prevented us from establishing potential specific cognitive signatures of individual CNVs.

In conclusion, CNVs are increasingly being identified in patients with neurodevelopmental disorders, including schizophrenia. The outcome of carrying a CNV can be mild and consistent with high levels of social and economic functioning. However, this varies according to the specific CNV involved, and certain rare neurodevelopmental CNVs are particularly associated with functional deficits that can amount to a full band lower on the various scales (e.g. one lower household income bracket), even among apparently healthy carriers. Patients and their families will want to learn more about their genetic condition and will be concerned about the impact on relatives who may have inherited the CNV. Psychiatrists will therefore be required to be fully informed about the range of outcomes possible in unaffected relatives. Clinicians who request CNV testing should first ensure that patients and their families receive appropriate pre- and post-test counselling. Finally, the fact that the CNVs studied can be associated with outcomes of such variable severity indicates the presence of modifying factors (genetic and environmental) that may be tractable to intervention. This is of profound interest and importance, and warrants future research.

## Supplementary material

For supplementary material accompanying this paper visit https://doi.org/10.1192/bjp.2018.301. click here to view supplementary material
