# Supplementary material for: Cognitive performance and functional outcomes of carriers of pathogenic copy number variants: analysis of the UK Biobank
Source: Br J Psychiatry. 2019 May;214(5):297–304. doi: 10.1192/bjp.2018.301 (PMC6520248; doi:10.1192/bjp.2018.301)
Supplement: Supplementary file 1 [file S000712501800301Xsup.zip › S000712501800301Xsup001.docx]

**Supplementary material**

**Supplementary Figure 1**. Effect size and 95% confidence intervals of the effect sizes for cognitive tests and functional outcomes.


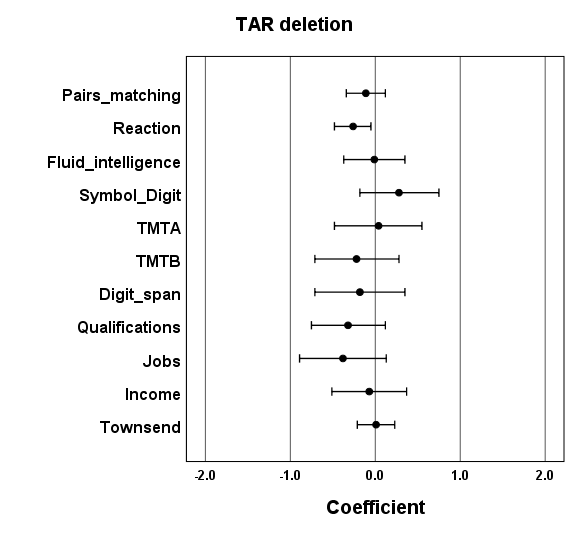

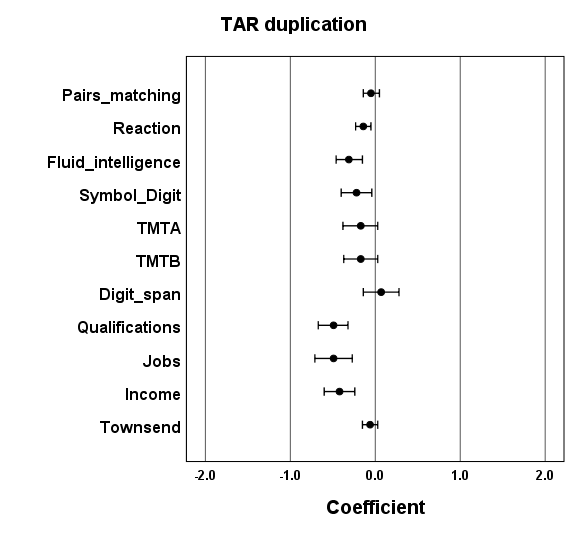


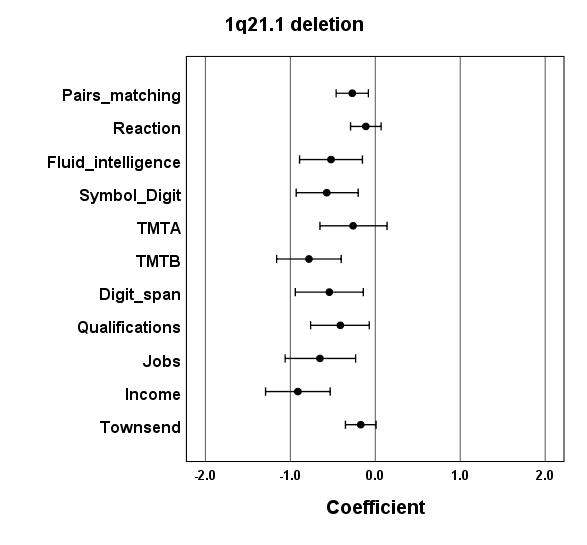

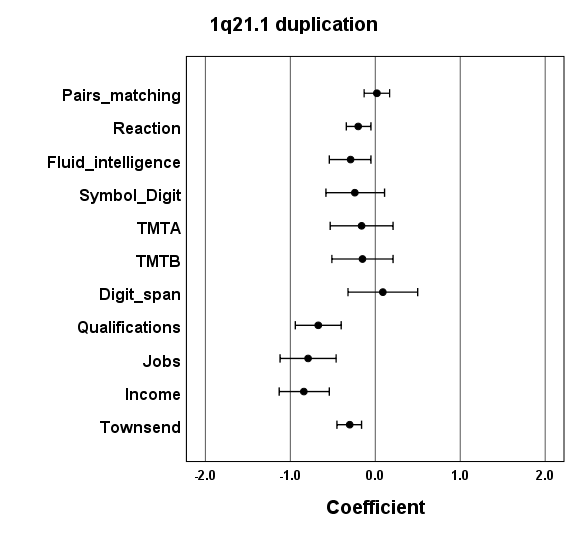


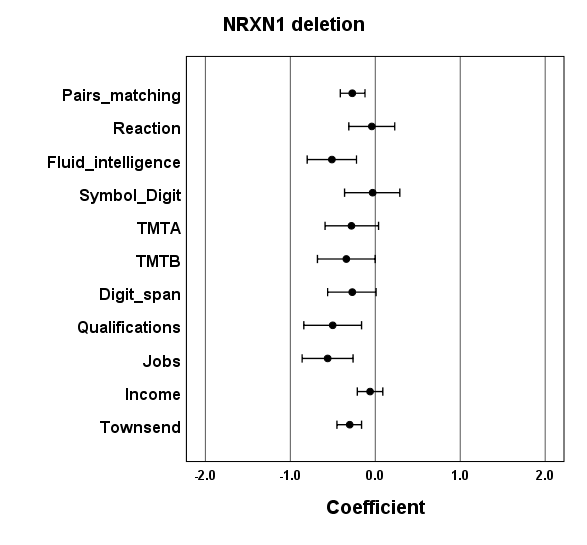


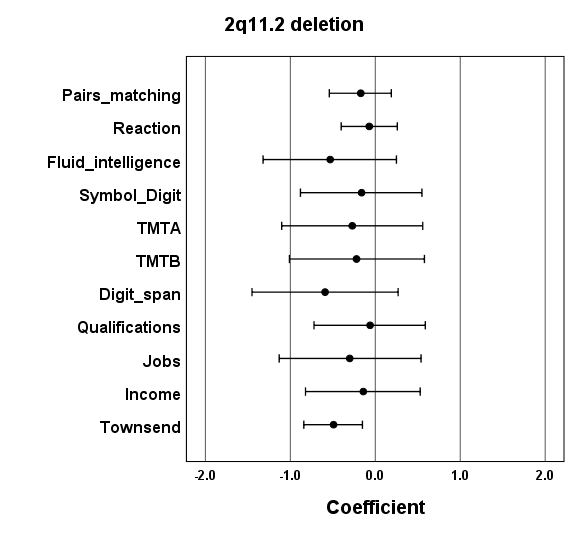


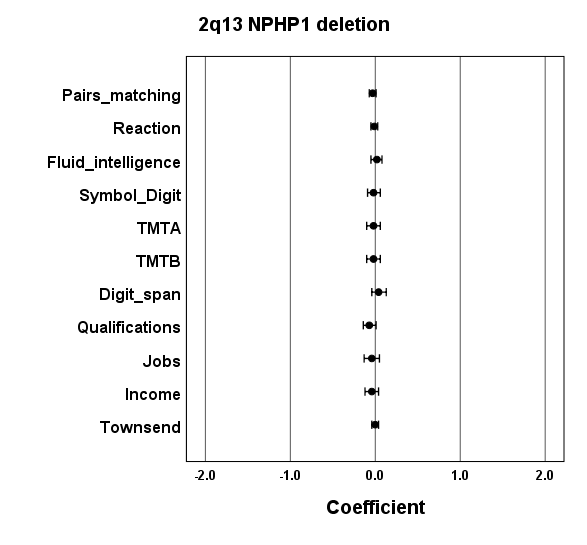

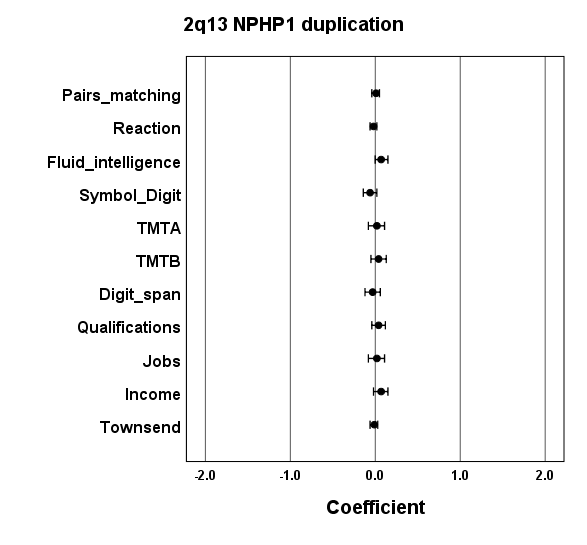


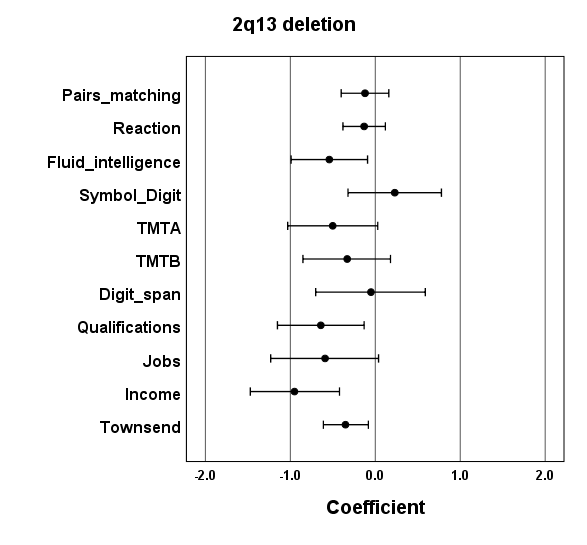

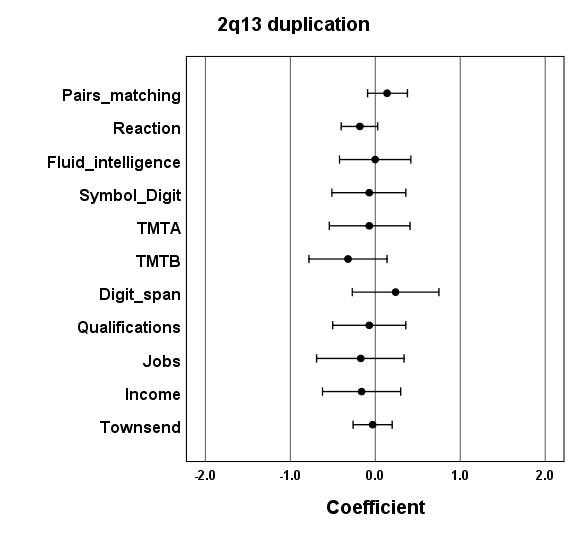


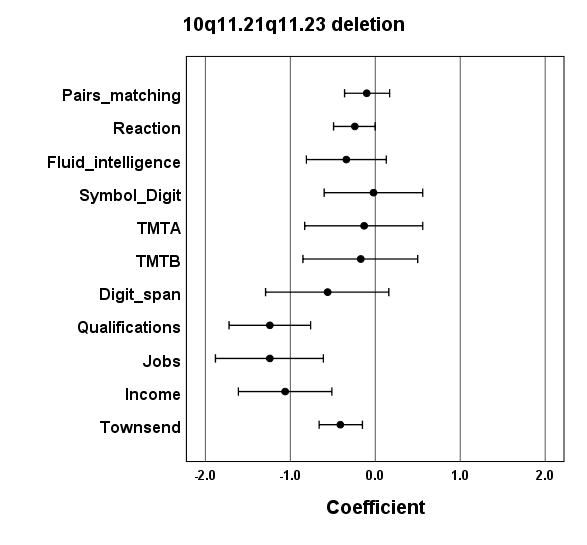

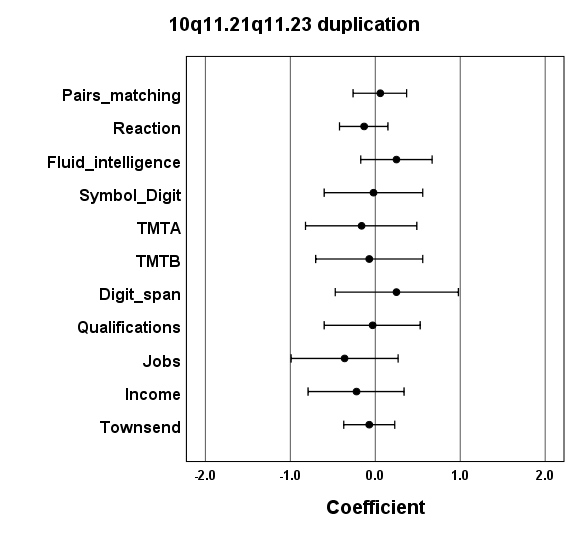


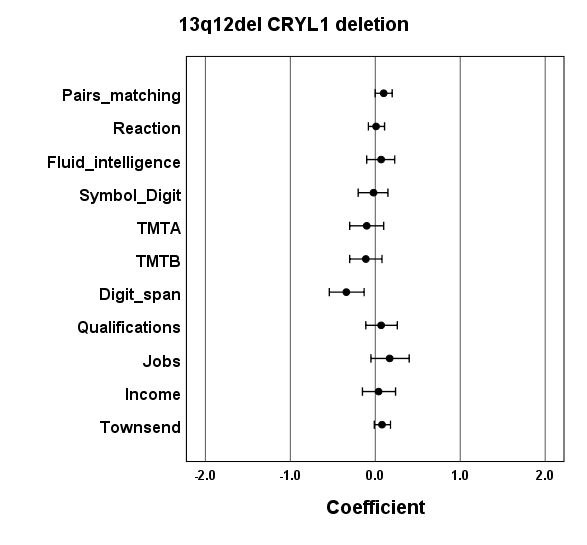


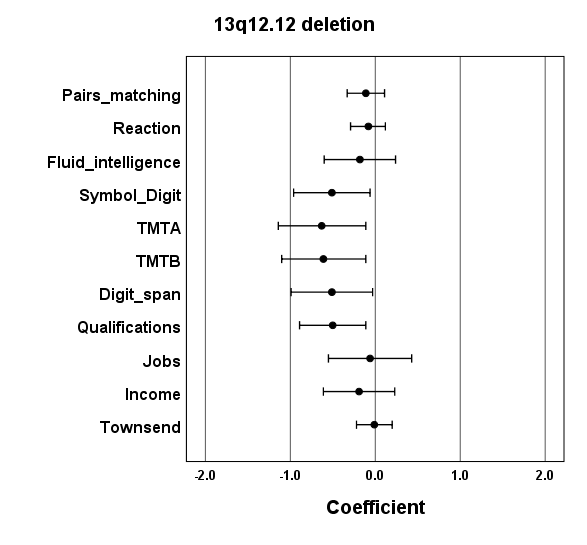

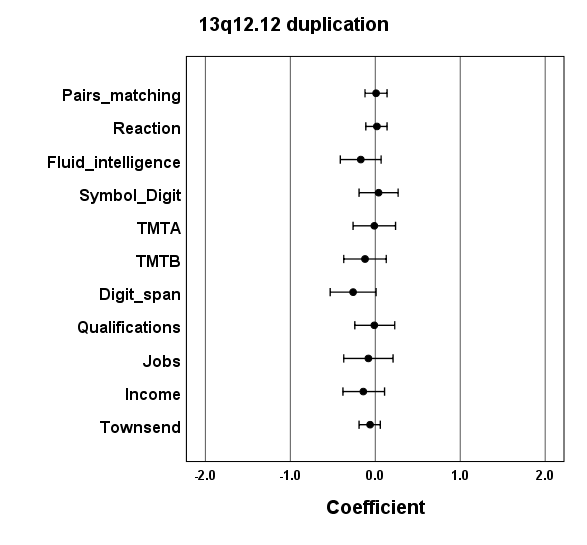


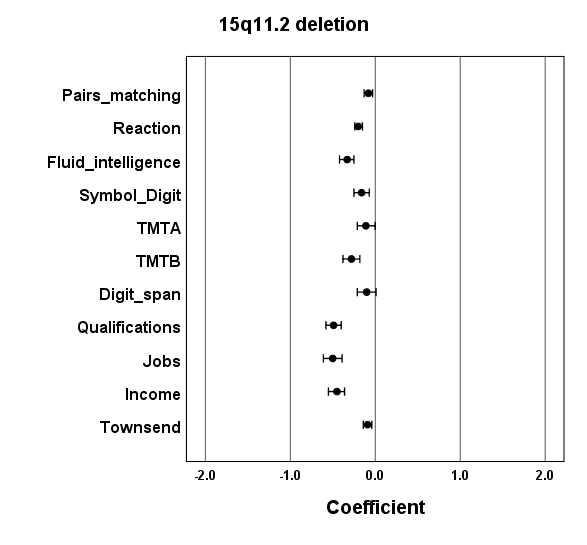

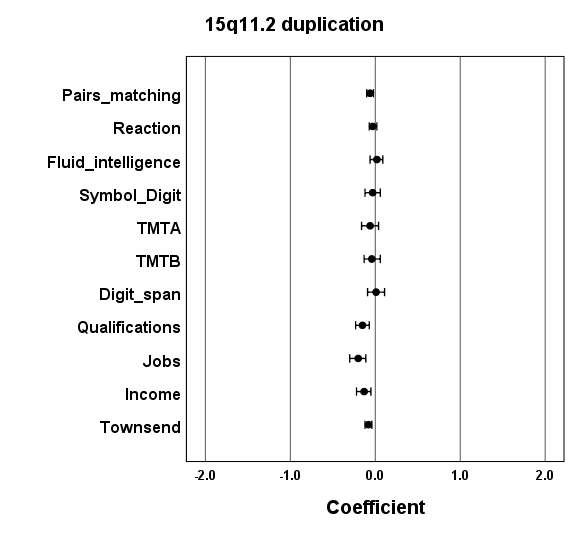


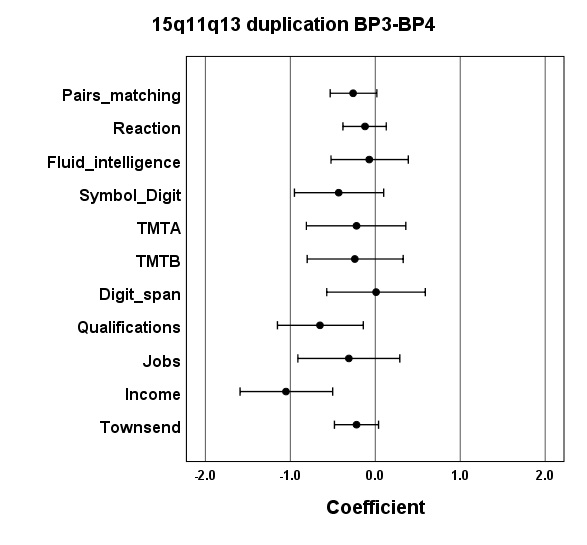


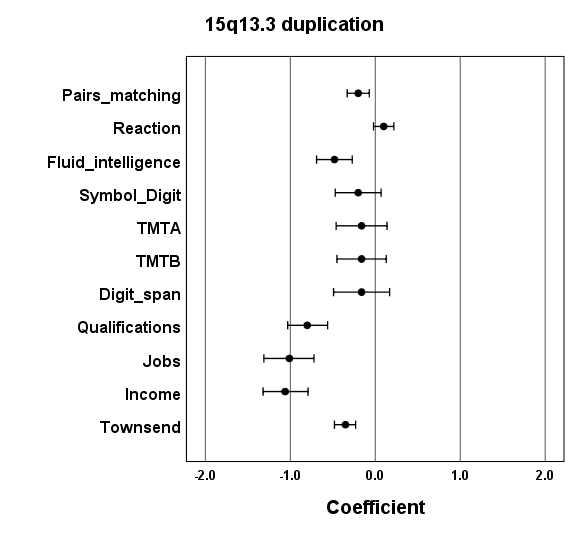


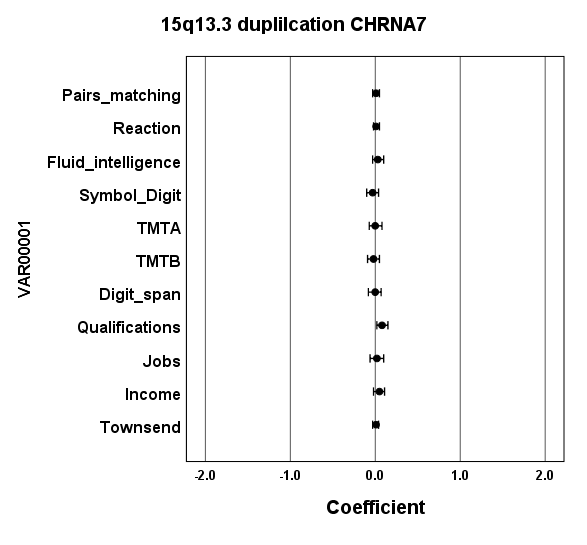


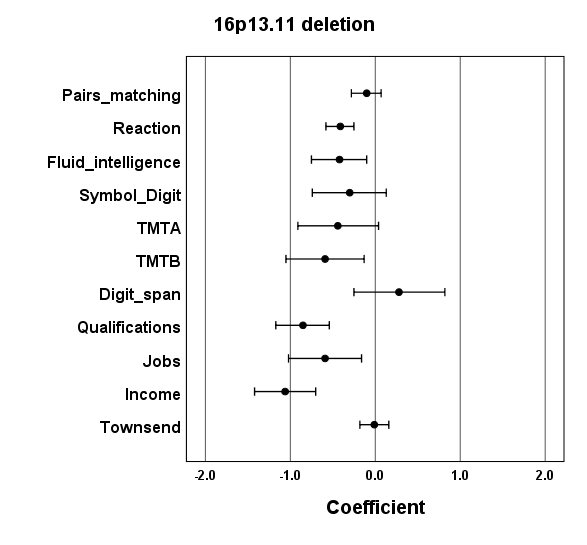

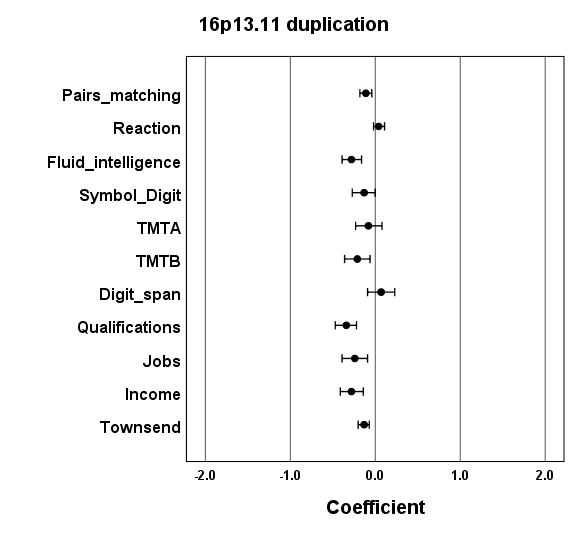


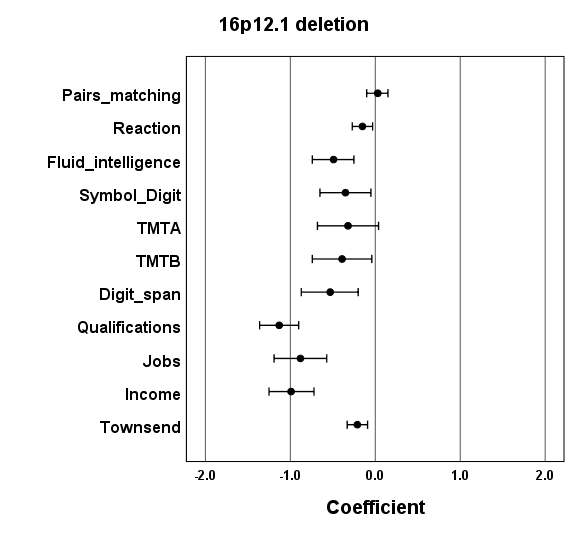

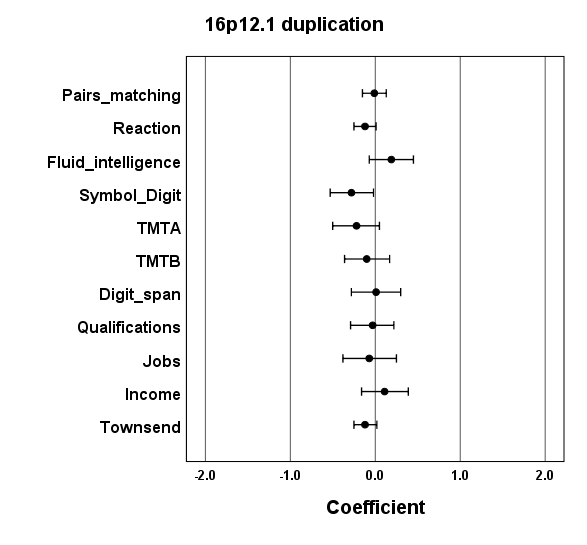


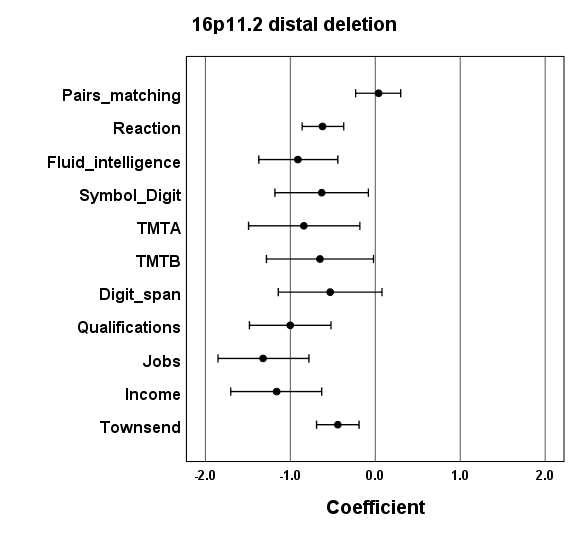

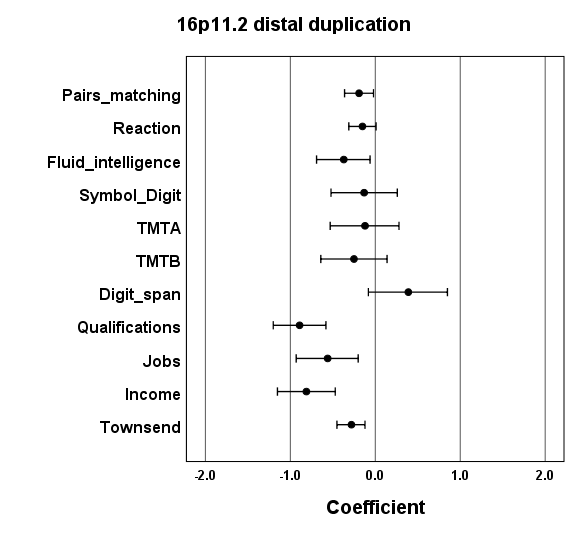


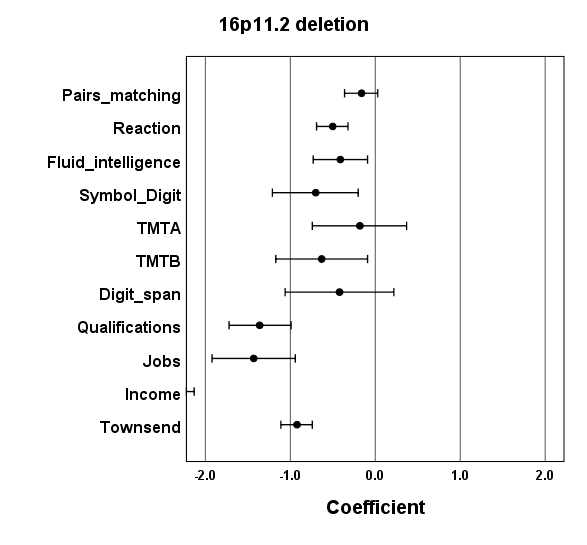

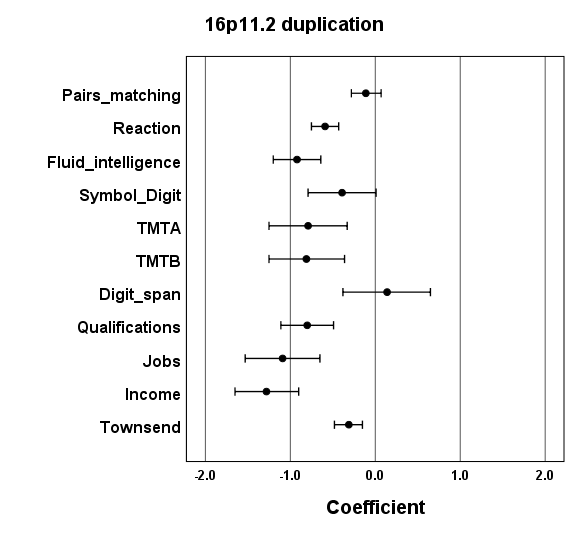


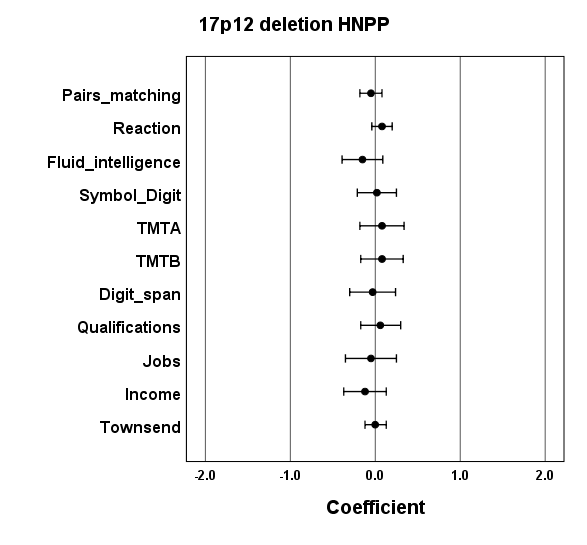

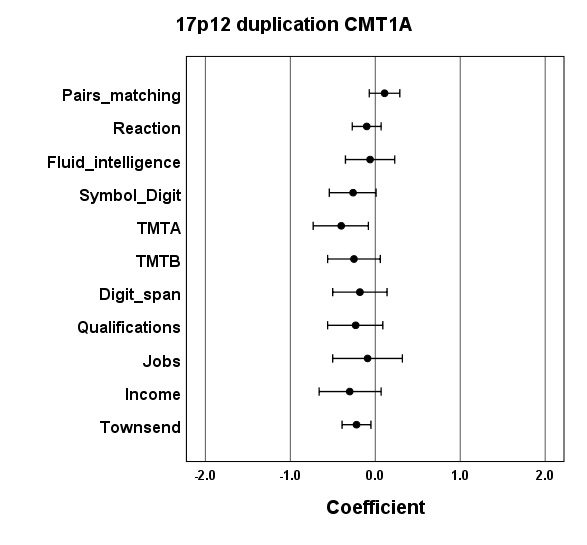


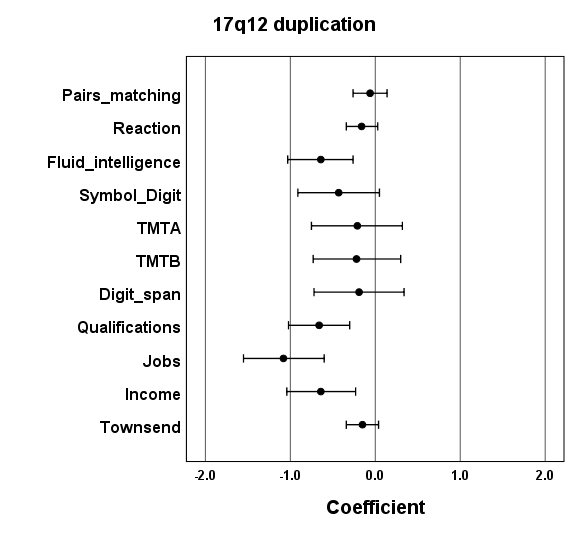


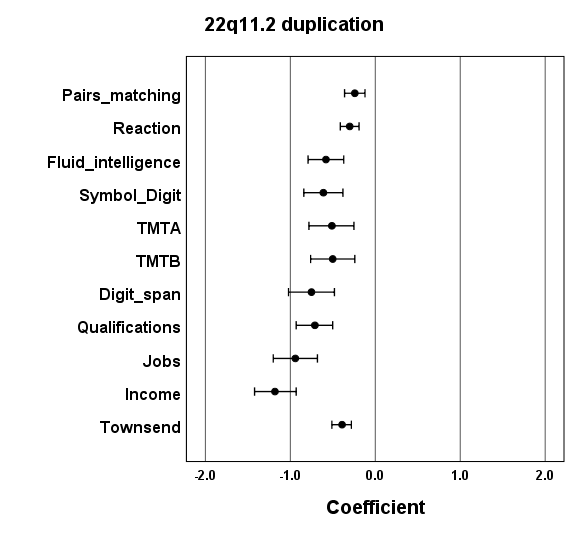


**Supplementary Table 2**. Differences in the average number of children that CNV carriers have, compared to the controls. Controls (participants without CNVs) have an average of 1.8 children. The values and corresponding p-values are produced from linear regression analysis after correction with age, sex and array type (nominally significant results in bold). A minus sign indicates fewer children. For completeness we provide the average effect size of the seven cognitive tests and the penetrance estimates. The number of children was truncated at six to bring the distribution closer to normality.

| CNV locus | Number of CNV carriers (%) | Average effect size | Penetrance (%) | Difference in number of children | p-value |
| --- | --- | --- | --- | --- | --- |
| *TAR del | 75 (0.018) | -0.11 | 17 | -0.04 | 0.76 |
| *TAR dup | 436 (0.1) | -0.12 | 7 | 0.03 | 0.60 |
| **1q21.1 del | 112 (0.027) | -0.27 | 28 | -0.59 | **9.9×10^-8^** |
| **1q21.1 dup | 176 (0.042) | -0.08 | 14 | 0.01 | 0.94 |
| ***NRXN1* del | 162 (0.039) | -0.19 | 13 | -0.25 | **0.008** |
| *2q11.2 del | 31 (0.007) | -0.13 | 13 | -0.22 | 0.3 |
| 2q13 del (*NPHP1*) | 2446 (0.58) | -0.01 | 4 | 0.03 | 0.19 |
| 2q13 dup (*NPHP1*) | 1972 (0.47) | 0.01 | 9 | 0.01 | 0.79 |
| *2q13 del | 53 (0.013) | -0.16 | 17 | -0.10 | 0.53 |
| *2q13 dup | 71 (0.017) | -0.02 | 6 | -0.34 | **0.016** |
| 2q21.1 dup | 59 (0.014) | 0.07 | 9 | -0.08 | 0.58 |
| *10q11.21q11.23 del | 57 (0.014) | -0.18 | 7 | -0.11 | 0.49 |
| 10q11.21q11.23 dup | 41 (0.01) | 0.03 | 11 | -0.37 | **0.045** |
| 13q12 del (*CRYL1*) | 379 (0.09) | 0.01 | 6 | 0.01 | 0.85 |
| 13q12.12 del | 84 (0.02) | -0.16 | 4 | 0.01 | 0.95 |
| 13q12.12 dup | 233 (0.055) | -0.01 | 3 | 0.15 | 0.051 |
| **15q11.2 del | 1661 (0.39) | -0.15 | 8 | -0.03 | 0.27 |
| *15q11.2 dup | 2039 (0.48) | -0.03 | 5 | -0.07 | **0.005** |
| 15q11q13 dup BP3-BP4 | 53 (0.013) | -0.18 | 4 | 0.10 | 0.54 |
| 15q13.3 dup | 240 (0.057) | -0.09 | 8 | -0.05 | 0.48 |
| 15q13.3 dup (*CHRNA7*) | 3023 (0.72) | 0.01 | 5 | 0.01 | 0.64 |
| *16p13.11 del | 131 (0.031) | -0.26 | 17 | -0.35 | **0.001** |
| **16p13.11 dup | 825 (0.2) | -0.06 | 7 | -0.03 | 0.41 |
| **16p12.1 del | 245 (0.058) | -0.10 | 11 | -0.03 | 0.71 |
| 16p12.1 dup | 198 (0.047) | -0.09 | 3 | -0.01 | 0.95 |
| *16p11.2 distal del | 58 (0.014) | -0.41 | 23 | -0.68 | **9.0×10^-6^** |
| *16p11.2 distal dup | 136 (0.032) | -0.20 | 11 | -0.21 | **0.041** |
| *16p11.2 del | 104 (0.025) | -0.34 | 38 | -1.0 | **3.3×10^-19^** |
| **16p11.2 dup | 134 (0.032) | -0.41 | 29 | -0.13 | 0.20 |
| 17p12 del (HNPP) | 237 (0.056) | 0.01 | 5 | -0.15 | 0.055 |
| 17p12 dup (CMT1A) | 124 (0.030) | -0.04 | 9 | 0.01 | 0.94 |
| *17q12 dup | 100 (0.024) | -0.18 | 14 | -0.06 | 0.62 |
| *22q11.2 dup | 279 (0.066) | -0.32 | 14 | -0.23 | **0.001** |

**Supplementary Table 3.** Effect of Fluid Intelligence on the strength of the correlations with the Townsend Deprivation Index, using linear regression. The two analyses were performed on participants who completed the Fluid Intelligence test, to ensure that they include identical sets of people, and therefore the results are not identical to those in the main text and tables. Some coefficients are weaker, indicating that the cognitive abilities examined by this test have an effect on the earning potential of the person. But there are still substantial effects remaining after correction for this test, indicating that there are other pathways that make CNV carriers end up living in areas with higher social deprivation.

| CNV | Original analysis | | | | Analysis controlled for Fluid Intelligence Score | | | |
| --- | --- | --- | --- | --- | --- | --- | --- | --- |
|  | p-value | B | 95% CI lower | 95% CI upper | p-value | B | 95% CI lower | 95% CI upper |
| TAR_del | 0.99 | 0.00 | 0.34 | -0.35 | 0.99 | 0.00 | -0.35 | 0.34 |
| TAR_dup | 0.020 | -0.18 | -0.03 | -0.32 | 0.058 | -0.14 | -0.29 | 0.01 |
| 1q21.1del | 0.84 | -0.04 | 0.32 | -0.39 | 0.95 | 0.01 | -0.34 | 0.36 |
| 1q21.1dup | 0.00021 | -0.45 | -0.21 | -0.68 | 0.00052 | -0.42 | -0.65 | -0.18 |
| NRXN1del | 0.77 | 0.04 | 0.30 | -0.22 | 0.73 | 0.05 | -0.21 | 0.31 |
| 2q11.2del | 4.3E-06 | -1.76 | -1.01 | -2.51 | 7.1E-06 | -1.71 | -2.46 | -0.97 |
| 2q13del_NPHP1 | 0.36 | -0.03 | 0.03 | -0.10 | 0.35 | -0.03 | -0.10 | 0.03 |
| 2q13dup_NPHP1 | 0.53 | 0.02 | 0.10 | -0.05 | 0.66 | 0.02 | -0.06 | 0.09 |
| 2q13del | 3.5E-05 | -0.92 | -0.48 | -1.35 | 9.9E-05 | -0.86 | -1.29 | -0.43 |
| 2q13dup | 0.57 | -0.12 | 0.28 | -0.52 | 0.58 | -0.11 | -0.51 | 0.29 |
| 2q21.1dup | 0.52 | -0.14 | 0.29 | -0.58 | 0.50 | -0.15 | -0.58 | 0.28 |
| 10q11.21q11.23del | 0.64 | -0.11 | 0.34 | -0.55 | 0.75 | -0.07 | -0.52 | 0.37 |
| 10q11.21q11.23dup | 0.65 | -0.09 | 0.31 | -0.49 | 0.57 | -0.11 | -0.51 | 0.28 |
| 13q12del_CRYL1 | 0.20 | 0.11 | 0.27 | -0.06 | 0.23 | 0.10 | -0.06 | 0.26 |
| 13q12.12del | 0.79 | -0.06 | 0.35 | -0.46 | 0.85 | -0.04 | -0.44 | 0.36 |
| 13q12.12dup | 0.47 | -0.08 | 0.14 | -0.31 | 0.56 | -0.07 | -0.29 | 0.16 |
| 15q11.2del | 0.036 | -0.09 | -0.01 | -0.17 | 0.18 | -0.06 | -0.14 | 0.03 |
| 15q11.2dup | 0.0044 | -0.11 | -0.03 | -0.18 | 0.0035 | -0.11 | -0.18 | -0.04 |
| 15q11q13dup_BP3_BP4 | 0.43 | 0.17 | 0.61 | -0.26 | 0.42 | 0.18 | -0.25 | 0.61 |
| 15q13.3dup | 2.1E-05 | -0.43 | -0.23 | -0.63 | 0.00014 | -0.39 | -0.58 | -0.19 |
| 15q13.3dup_CHRNA7 | 0.32 | -0.03 | 0.03 | -0.09 | 0.27 | -0.03 | -0.09 | 0.03 |
| 16p13.11del | 0.80 | -0.04 | 0.27 | -0.35 | 0.97 | 0.01 | -0.30 | 0.32 |
| 16p13.11dup | 0.0065 | -0.16 | -0.04 | -0.27 | 0.022 | -0.13 | -0.24 | -0.02 |
| 16p12.1del | 0.016 | -0.29 | -0.05 | -0.52 | 0.046 | -0.24 | -0.47 | 0.00 |
| 16p12.1dup | 0.13 | -0.19 | 0.06 | -0.44 | 0.10 | -0.21 | -0.45 | 0.04 |
| 16p11.2distal_del | 0.00025 | -0.83 | -0.39 | -1.28 | 0.0011 | -0.74 | -1.19 | -0.30 |
| 16p11.2distal_dup | 0.020 | -0.35 | -0.05 | -0.65 | 0.035 | -0.32 | -0.62 | -0.02 |
| 16p11.2del | 1.3E-05 | -0.68 | -0.38 | -0.99 | 3.9E-05 | -0.64 | -0.95 | -0.33 |
| 16p11.2dup | 0.00052 | -0.47 | -0.20 | -0.74 | 0.0049 | -0.38 | -0.64 | -0.11 |
| 17p12del_HNPP | 0.72 | -0.04 | 0.19 | -0.27 | 0.80 | -0.03 | -0.26 | 0.20 |
| 17p12dup_CMT1A | 0.0018 | -0.44 | -0.16 | -0.72 | 0.0021 | -0.43 | -0.71 | -0.16 |
| 17q12dup | 0.70 | -0.07 | 0.30 | -0.44 | 0.97 | -0.01 | -0.37 | 0.36 |
| 22q11.2dup | 0.00010 | -0.40 | -0.20 | -0.61 | 0.00077 | -0.35 | -0.55 | -0.14 |

**Supplementary Table 4.** Effect of Fluid Intelligence on the strength of the correlations with the Household Income, using ordinal regression. The two analyses were performed on participants who completed the Fluid Intelligence test, to ensure that they include identical sets of people, and therefore the results are not identical to those in the main text and tables. Most estimates (effect sizes) are reduced, indicating that the cognitive abilities examined by this test have an effect on the earning potential of the person. But there are still substantial effects remaining after correction for this test, indicating that there are other pathways that make CNV carriers end up earning a lower income.

| CNV | Original analysis | | | | Analysis controlled for Fluid Intelligence Score | | | |
| --- | --- | --- | --- | --- | --- | --- | --- | --- |
|  | p-value | B | 95% CI lower | 95% CI upper | p-value | B | 95% CI lower | 95% CI upper |
| TAR_del | 0.72 | -0.13 | -0.82 | 0.56 | 0.75 | -0.11 | -0.81 | 0.58 |
| TAR_dup | 0.00015 | -0.60 | -0.92 | -0.29 | 0.0054 | -0.44 | -0.76 | -0.13 |
| 1q21.1del | 0.10 | -0.60 | -1.33 | 0.12 | 0.39 | -0.32 | -1.04 | 0.41 |
| 1q21.1dup | 0.00051 | -0.89 | -1.39 | -0.39 | 0.0018 | -0.81 | -1.32 | 0.30 |
| NRXN1del | 0.082 | -0.46 | -0.98 | 0.59 | 0.12 | -0.41 | -0.94 | 0.11 |
| 2q11.2del | 0.85 | -0.15 | -1.72 | 1.42 | 0.97 | 0.026 | -1.55 | 1.61 |
| 2q13del_NPHP1 | 0.79 | -0.02 | -0.15 | 0.12 | 0.87 | -0.01 | -0.15 | 0.12 |
| 2q13dup_NPHP1 | 0.16 | 0.11 | -0.04 | 0.26 | 0.34 | 0.07 | -0.08 | 0.22 |
| 2q13del | 5.1E-07 | -2.91 | -4.04 | -1.78 | 8.4E-0.6 | -2.53 | -3.64 | -1.42 |
| 2q13dup | 0.67 | -0.19 | -1.08 | 0.69 | 0.57 | -0.26 | -1.16 | 0.63 |
| 2q21.1dup | 0.83 | 0.09 | -0.76 | 0.94 | 0.77 | 0.13 | -0.73 | 0.98 |
| 10q11.21q11.23del | 0.14 | -0.69 | -1.61 | 0.23 | 0.26 | -0.53 | -1.46 | 0.40 |
| 10q11.21q11.23dup | 0.85 | -0.08 | -0.87 | 0.72 | 0.95 | 0.02 | -0.76 | 0.81 |
| 13q12del_CRYL1 | 0.22 | 0.21 | -0.13 | 0.55 | 0.26 | 0.20 | -0.14 | 0.54 |
| 13q12.12del | 0.50 | -0.29 | -1.15 | 0.56 | 0.74 | -0.15 | -1.01 | 0.73 |
| 13q12.12dup | 0.34 | -0.22 | -0.68 | 0.34 | 0.57 | -0.13 | -0.59 | 0.33 |
| 15q11.2del | 1.5E-05 | -0.38 | -0.55 | -0.21 | 0.018 | -0.21 | -0.38 | 0.04 |
| 15q11.2dup | 0.19 | -0.10 | -0.25 | 0.05 | 0.12 | -0.12 | -0.27 | 0.03 |
| 15q11q13dup_BP3_BP4 | 0.54 | -0.28 | -1.16 | 0.60 | 0.50 | -0.31 | -1.20 | 0.58 |
| 15q13.3dup | 0.00015 | -0.83 | -1.25 | -0.40 | 0.005 | -0.62 | -1.05 | -0.19 |
| 15q13.3dup_CHRNA7 | 0.043 | 0.13 | 0.00 | 0.25 | 0.067 | 0.12 | -0.01 | 0.24 |
| 16p13.11del | 0.00031 | -1.27 | -1.96 | -0.58 | 0.0021 | -1.08 | -1.78 | -0.39 |
| 16p13.11dup | 0.049 | -0.24 | -0.48 | 0.00 | 0.45 | -0.09 | -0.33 | 0.15 |
| 16p12.1del | 0.0028 | -0.75 | -1.23 | -0.26 | 0.041 | -0.51 | -1.01 | -0.02 |
| 16p12.1dup | 0.34 | -0.25 | -0.77 | 0.27 | 0.15 | -0.38 | -0.90 | 0.14 |
| 16p11.2distal_del | 0.0043 | -1.50 | -2.54 | -0.47 | 0.047 | -1.07 | -2.12 | -0.01 |
| 16p11.2distal_dup | 0.051 | -0.61 | -1.23 | 0.00 | 0.13 | -0.49 | -1.11 | 0.14 |
| 16p11.2del | 7.7E-11 | -2.46 | -3.20 | -1.72 | 1.3E-09 | -2.31 | -3.06 | -1.57 |
| 16p11.2dup | 3.4E-09 | -1.89 | -2.51 | -1.26 | 3.7E-06 | -1.49 | -2.12 | -0.86 |
| 17p12del_HNPP | 0.049 | -0.46 | -0.92 | 0.00 | 0.081 | -0.41 | -0.88 | 0.051 |
| 17p12dup_CMT1A | 0.67 | -0.13 | -.73 | 0.47 | 0.92 | -0.03 | -0.63 | 0.67 |
| 17q12dup | 0.23 | -0.47 | -1.22 | 0.29 | 0.59 | -0.21 | -0.97 | 0.55 |
| 22q11.2dup | 1.9E-08 | -1.27 | -1.72 | -0.83 | 1.0E-5 | -1.01 | -1.45 | -0.56 |

**Supplementary Table 5.** Bivariate Pearson correlations of the seven cognitive tests and the four measures of functioning among controls. All are positively correlated with each other and all (but one) are significant. Top half: correlations among controls; bottom half: correlations among CNV carriers (excluding the five most common CNVs, as they behave similarly to controls (deletions and duplications at 15q11.2, NPHP1 and duplications at CHRNA7)). The correlations are quite similar between the two groups. Directions of effect are corrected, so that a positive sign always indicates that worse performance correlates with worse performance.

|  | | Reaction time | Pairs matching | Fluid Intelligence | Digit span | TMTA | TMTB | Symbol Digit | Jobs | Qualifications | Income household | Townsend |
| --- | --- | --- | --- | --- | --- | --- | --- | --- | --- | --- | --- | --- |
| Reaction time | Correlation |  | .120 | .161 | .076 | .204 | .248 | .257 | .088 | .162 | .210 | .052 |
|  | Sig. (2-tailed) |  | 0.E+00 | 0.E+00 | 2.E-119 | 0.E+00 | 0.E+00 | 0.E+00 | 0.E+00 | 0.E+00 | 0.E+00 | 1.E-245 |
| Pairs matching | Correlation | .094 |  | .176 | .084 | .128 | .196 | .186 | .049 | .081 | .085 | .004 |
|  | Sig. (2-tailed) | 2.8E-11 |  | 0.E+00 | 2.E-144 | 0.E+00 | 0.E+00 | 0.E+00 | 6.E-139 | 0.E+00 | 0.E+00 | .014 |
| Fluid Intelligence | Correlation | .168 | .148 |  | .292 | .219 | .388 | .293 | .302 | .466 | .281 | .093 |
|  | Sig. (2-tailed) | 2.3E-11 | 3.5E-09 |  | 0.E+00 | 0.E+00 | 0.E+00 | 0.E+00 | 0.E+00 | 0.E+00 | 0.E+00 | 2.E-253 |
| Digit span | Correlation | .053 | .066 | .198 |  | .200 | .309 | .217 | .126 | .190 | .145 | .005 |
|  | Sig. (2-tailed) | .12 | 0.049 | .0003 |  | 0.E+00 | 0.E+00 | 0.E+00 | 7.E-221 | 0.E+00 | 0.E+00 | .137 |
| TMTA | Correlation | .225 | .055 | .168 | .188 |  | .660 | .509 | .137 | .167 | .220 | .010 |
|  | Sig. (2-tailed) | 1.2E-11 | 0.1 | .002 | 1.2E-07 |  | 0.E+00 | 0.E+00 | 3.E-253 | 0.E+00 | 0.E+00 | .004 |
| TMTB | Correlation | .206 | .133 | .320 | .320 | .651 |  | .580 | .170 | .264 | .267 | .020 |
|  | Sig. (2-tailed) | 5.9E-10 | .00007 | 2.0E-09 | 4.4E-20 | 3.7E-108 |  | 0.E+00 | 0.E+00 | 0.E+00 | 0.E+00 | 1.E-09 |
| Symbol Digit | Correlation | .256 | .137 | .250 | .240 | .517 | .569 |  | .161 | .232 | .267 | .024 |
|  | Sig. (2-tailed) | 3.2E-16 | .00002 | 1.0E-06 | 9.3E-13 | 2.2E-60 | 2.9E-75 |  | 0.E+00 | 0.E+00 | 0.E+00 | 5.E-14 |
| Jobs | Correlation | .080 | .063 | .363 | .131 | .168 | .195 | .234 |  | .470 | .414 | .160 |
|  | Sig. (2-tailed) | .000016 | 0.001 | 2.5E-37 | .001 | .00004 | 1.5E-06 | 1.2E-09 |  | 0.E+00 | 0.E+00 | 0.E+00 |
| Qualifications | Correlation | .170 | .043 | .475 | .170 | .147 | .276 | .224 | .524 |  | .426 | .155 |
|  | Sig. (2-tailed) | 5.2E-32 | .003 | 1.0E-84 | 8.2E-07 | .00002 | 0.E+00 | 4.3E-12 | 8.9E-198 |  | 0.E+00 | 0.E+00 |
| Income household | Correlation | .218 | .096 | .329 | .106 | .242 | .277 | .277 | .438 | .440 |  | .229 |
|  | Sig. (2-tailed) | 1.3E-45 | 4.8E-10 | 6.4E-35 | .003 | 3.3E-12 | 1.1E-15 | 3.9E-17 | 3.4E-123 | 8.8E-188 |  | 0.E+00 |
| Townsend | Correlation | .078 | .018 | .230 | .009 | .100 | .066 | .055 | .226 | .237 | .325 |  |
|  | Sig. (2-tailed) | 3.2E-08 | .2 | 2.3E-20 | .79 | .003 | .048 | .083 | 1.3E-35 | 9.0E-62 | 1.5E-103 |  |

**Supplementary Table 6. Schizophrenia-associated CNVs and measures of functioning.** The table provides the details for Table 3 in the main text. Shown are the unstandardized coefficients (B) and 95% confidence intervals (95% CI) of the B from linear regression analyses (for the Townsend Deprivation Index), and the estimates coefficients from ordinal regression analyses (for qualifications, occupation and income).

| **CNV** | **Qualifications** | **Occupation** | **Household income** | **Townsend index** |
| --- | --- | --- | --- | --- |
| 1q21.1 del | -0.41 (-0.76 -0.07) | -0.65 (-1.06 -0.23) | -0.91 (-1.29 -0.53) | -0.21 (-0.39 -0.03) |
| 1q21.1 dup | -0.67 (-0.94 -0.4) | -0.79 (-1.12 -0.46) | -0.84 (-1.13 -0.54) | -0.38 (-0.52 -0.23) |
| NRXN1 del | -0.27 (-0.56 0.01) | -0.5 (-0.84 -0.16) | -0.56 (-0.86 -0.26) | -0.1 (-0.25 0.06) |
| 3q29 del | -0.17 (-1.59 1.25) | -0.32 (-2.03 1.4) | -2.44 (-4.08 -0.79) | -1.03 (-1.76 -0.29) |
| WBS dup | -1.45 (-2.55 -0.36) | -2.16 (-3.31 -1) | -2.61 (-4.22 -1.01) | -0.57 (-1.11 -0.03) |
| 15q11.2 del | -0.49 (-0.58 -0.4) | -0.5 (-0.61 -0.39) | -0.45 (-0.55 -0.36) | -0.1 (-0.15 -0.05) |
| PWS dup | -1.51 (-2.41 -0.62) | -2.33 (-3.38 -1.28) | -1.61 (-2.63 -0.59) | -0.61 (-1.08 -0.14) |
| 15q13.3 del | -1.09 (-1.65 -0.53) | -1.63 (-2.34 -0.91) | -1.58 (-2.21 -0.95) | -1.02 (-1.32 -0.72) |
| 16p13.11 dup | -0.34 (-0.47 -0.22) | -0.24 (-0.39 -0.09) | -0.28 (-0.41 -0.14) | -0.15 (-0.21 -0.08) |
| 16p12.1 del | -1.13 (-1.36 -0.9) | -0.88 (-1.19 -0.57) | -0.99 (-1.25 -0.72) | -0.27 (-0.4 -0.15) |
| 16p11.2 dup | -0.8 (-1.11 -0.49) | -1.09 (-1.53 -0.65) | -1.28 (-1.65 -0.9) | -0.4 (-0.57 -0.23) |
| 22q11.2 del | -1.96 (-3.28 -0.63) | -3.5 (-5.23 -1.77) | -3.4 (-5.62 -1.17) | -1.22 (-1.91 -0.54) |
